# Knee Pathology before and after SARS-CoV-2 Pandemic: An Analysis of 1139 Patients

**DOI:** 10.3390/healthcare9101311

**Published:** 2021-09-30

**Authors:** Riccardo Giorgino, Davide Maria Maggioni, Marco Viganò, Fabio Verdoni, Elisa Pandini, Cristiana Balbino, Nicola Manta, Roberto D’Anchise, Laura Mangiavini

**Affiliations:** 1 Residency Program in Orthopedics and Traumatology, University of Milan, 20122 Milan, Italy; riccardo.giorgino@unimi.it (R.G.); davidemaria.maggioni@unimi.it (D.M.M.); 2 IRCCS Istituto Ortopedico Galeazzi, 20161 Milan, Italy; marco.vigano@grupposandonato.it (M.V.); fabio.verdoni@grupposandonato.it (F.V.); elisa.pandini@grupposandonato.it (E.P.); cristiana.balbino@grupposandonato.it (C.B.); nicola.manta@grupposandonato.it (N.M.); roberto.danchise@grupposandonato.it (R.D.); 3 Department of Biomedical Sciences for Health, University of Milan, 20122 Milan, Italy

**Keywords:** COVID-19, knee, patellofemoral pain syndrome, SARS-CoV-2 pandemic, lockdown

## Abstract

**Simple Summary:**

The SARS-CoV-2 pandemic drastically changed daily life activities and medical practice and a total reorganization of healthcare activities was required. People changed their daily habits and undertook a more sedentary lifestyle, leading to significant consequences in knee pathologies. The aim of this study was to evaluate the outpatient activity for knee pathologies before and after lockdown, in order to understand better which kind of pathologies orthopedic field is going to face. Further studies are required to validate this result with more in-depth clinical and radiological elements and to evaluate any differences in surgical treatment.

**Abstract:**

Background: The SARS-CoV-2 pandemic drastically changed daily life activities and medical practice, leading to a reorganization of healthcare activities. People spent two months in home-isolation, changing their daily habits and undertaking a more sedentary lifestyle. Change in lifestyle is related to important consequences in knee pathologies. The aim of this study was to evaluate the outpatient activity for knee pathologies before and after lockdown in terms of incidence, severity, diagnosis, and treatment. Methods: Medical records of patients with knee pathology in outpatient follow-up at IRCCS Istituto Ortopedico Galeazzi in Milan (Italy) were analyzed in the time frame 4 May–4 September 2020 and compared with patients examined between 4 May and 4 September 2019. Results: A significant increase of knee diagnoses associated to patellofemoral disorders in 2020 was found (*p* = 0.004). In addition, physiotherapy was significantly more prescribed in 2020 than in 2019 (*p* = 0.012). Conclusions: The SARS-CoV-2 pandemic lockdown did not drastically change knee pathology, but it may have had an impact on it, highlighting a summary worsening of patellofemoral disorders associated with other knee diagnoses. Further studies are required to validate this result.

## 1. Introduction

After the spread of the SARS-CoV-2 pandemic around the world, daily life activities and medical practice have drastically changed [1,2,3]. During the first pandemic wave, Italy was one of the most affected countries and Italian hospitals have been deeply involved in the management of patients affected by COVID-19 infection and other comorbidities with the need for a reorganization of healthcare activities [4]. To reduce the spread of COVID-19 in the population, several isolation measures were adopted, and a national lockdown was established, significantly limiting all non-essential activities [3]. Since 10 March 2020, Italy was in lockdown: all the commercial activities were closed, travels were forbidden, and social interactions were discouraged. According to the trend of the pandemic in Italy, the National Government declared that on 4 May Italy moved to “phase 2”, the first real step towards the reopening, allowing reunions among relatives and individual recreational activity at a distance. The reopening of the retail trade instead was scheduled for 18 May 2020. Therefore, in our hospital, we adjusted healthcare activities considering the particular historical moment and a reorganization of the assistance activities was mandatory [2,4,5]. During the March–April 2020 lockdown, our Regional Health System was reorganized following the Hub–Spoke model [6,7]. In this context, our institution was identified as a hub for ‘minor’ trauma, defined as low-energy, single-district trauma requiring an orthopedic surgical treatment [5]. All outpatient and elective surgery activities were temporally suspended until Italy moved to “phase 2”. Due to the limitations, people spent two months in home-isolation, changing their daily habits and undertaking a more sedentary lifestyle than usual [8]. Change in lifestyle is related to important consequences in orthopedic pathologies and in particular joint pathologies, such as the knee [9,10,11,12]. In the months following the outbreak of the first pandemic wave of March–April 2020, the outpatient activity of our hospital resumed, albeit with different rhythms and care services, dealing with an accumulation of untreated patients. To maintain social distancing within the building, a filter system was created at the entrance to minimize the number of people inside the hospital. In addition, the time scheduled for the single visit was increased. The aim of this study was to evaluate the outpatient activity for knee pathologies after lockdown in terms of incidence, severity, diagnosis, and treatment and to compare it to the outpatient activity in the same timeframe in 2019.

## 2. Materials and Methods

Medical records of patients with knee pathology in outpatient follow-up at IRCCS Istituto Ortopedico Galeazzi in Milan (Italy) were analyzed in the timeframe 4 May 2020–4 September 2020 and compared with patients examined between 4 May–4 September 2019. We selected a four-month timeframe following 55 days of national lockdown. Data of each patient were collected from the medical records, such as age, sex, laterality, diagnosis, requested radiological examinations, and treatment. The principal requirement for inclusion was a diagnosis of knee pathology. All diagnoses were assessed by two orthopedic surgeons, on the basis of (i) the history of present disease, (ii) a physical examination, (iii) imaging (if present). Diagnoses were collected and grouped according to the etiopathogenesis (Table 1).

The prescription of additional imaging was highlighted and grouped as follow: need for (i) Rx, (ii) MRI, (iii) CT scan, and (iv) scintigraphy.

The treatment choice was grouped: (i) home exercises to perform independently, such as isometric strengthening of quadriceps, and suggested physical activity, such as cycling or swimming, (ii) drug therapy, and (iii) physical therapy, such as (1) magneto therapy, (2) tecar therapy, (3) magneto therapy + tecar therapy, (4) laser therapy, (5) shock-absorbing waves, (iv) physiotherapy, and (v) surgery.

### 2.1. Population Characteristics

**Figure 1:**
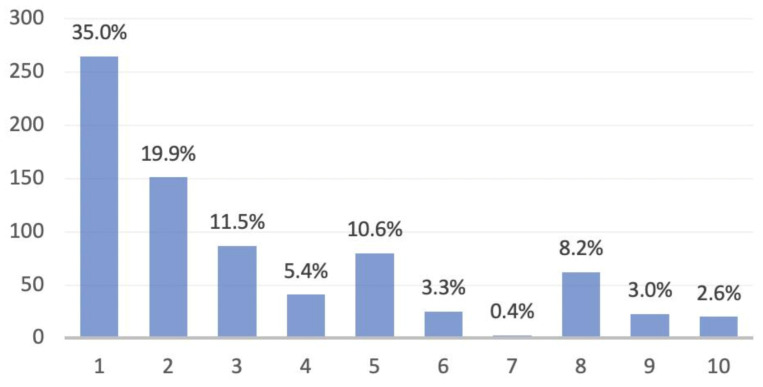
Distribution of the ten groups of diagnoses in 2019 knee medical records. (1) Post-surgery follow-up. (2) Degenerative disease. (3) Meniscus pathology. (4) Ligamentous pathology. (5) Patellofemoral disorders. (6) Osteocartilage pathology. (7) Growth pathology. (8) Traumatic injuries. (9) Other causes. (10) Unknown causes.

**Figure 2:**
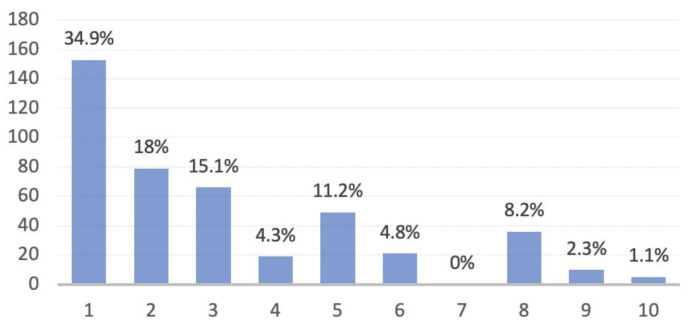
Distribution of the ten groups of diagnoses in 2020 knee medical records. (1) Post-surgery follow-up. (2) Degenerative disease. (3) Meniscus pathology. (4) Ligamentous pathology. (5) Patellofemoral disorders. (6) Osteocartilage pathology. (7) Growth pathology. (8) Traumatic injuries. (9) Other causes. (10) Unknown causes.

We collected a series of 1139 consecutive medical records. Of these, 726 were accomplished in 2019, while 413 were carried out in 2020. The diagnoses were distributed as represented in Figure 1 and Figure 2. The mean age of the population was 52 ± 20.27(SD) in 2019 and 52 ± 19.02(SD) in 2020. Considering sex, 341 males and 385 females underwent medical examination in 2019, while in 2020 there were 214 males and 199 females.

### 2.2. Statistical Analyses

The analysis was performed using R software v4.0.3 (R Core Team, Wien, Austria). Counts and percentage have been used to describe the categorical variables in the two groups, while mean and standard deviation were used to report continuous variables. Fisher’s exact test was used to assess the differences between groups in terms of categorical variables, unpaired t tests were applied for numerical ones. Shapiro–Wilk test was used to confirm normal data distribution. *p* values < 0.05 were considered statistically significant.

## 3. Results

Considering all the 1139 medical records, we did not find significant statistical differences between the 2019 and 2020 groups according to age (*p* = 0.939), sex (*p* = 0.123), diagnoses groups (*p* = 0.280), need for additional imaging (*p* = 0.146). Focusing on patients with patellofemoral disorders only (diagnosis group 5), we did not find significant difference in the incidence between 2019 and 2020, but we found a significant increase of patients with associated diagnoses (additional to patellofemoral disorders) diagnosed in 2020 compared to those diagnosed in 2019 (*p* = 0.004). Specifically, the pathologies associated to the patellofemoral disorders were degenerative disease, meniscus pathology, and osteocartilage pathology (Table 2). In addition, considering the patellofemoral disorders, we did not find a significant difference in terms of sex, age, need for additional imaging or the different conservative treatments, except for physiotherapy, which was significantly more prescribed in 2020 than in 2019 (20.7% in 2020; 2.2% in 2019; *p* = 0.012) (Table 2).

## 4. Discussion

According to the main findings of the present study, other knee pathologies resulted more frequently associated with patellofemoral pain syndrome (PFPS) in 2020 than in 2019. Furthermore, physiotherapy indication was also more frequent in 2020 than in 2019. Our results partially reflect our initial hypothesis, namely that the SARS-CoV-2 pandemic lockdown may have triggered or worsened PFPS. The rationale behind our reasoning lays in the possible consequences of a global lockdown: that is the reduction of sport and physical activities.

Significant scientific findings have already shown how this aspect plays a key role in the onset of PFPS. Literature has shown how an exact cause is still unknown, but a multifactorial etiology is probably responsible for this syndrome, which generally affects young women [13,14,15]. Biomechanical alterations in axial defects and quadriceps femoral muscle imbalances were found as common causes. The restrictive measures adopted during the lockdown led to a sedentary lifestyle [8] for the majority of the population, most likely leading to a reduction in muscle tone [16]. Indeed, other authors already provided recommendations on how to manage physical activity during COVID-19 induced lockdown [17]. The reduction of the trophism of some muscles such as the vastus medialis obliquus (VMO) had already been identified as associated to the PFPS [18]. Cowan et al. studied VMO size in PFPS through MRI, demonstrating that its atrophy is a contributing factor in PFPS, probably playing a role in the mechanism of abnormal patellar tracking [19]. Other studies have shown that alterations in the hip musculature may also be responsible for a functional malalignment responsible for PFPS. Indeed, several papers highlighted a reduction in the relative abduction force of the hip, in the external rotators of the hip, and in extension strength in patients with PFPS [20,21,22].

Another interesting aspect is the possible correlation between the psychologic aspect and the onset of the syndrome. Numerous studies have shown that a mutation in the mental state of the subjects occurred during the lockdown [23,24,25]. The psychological aspect plays an important role in PFPS that should not be underestimated. In a descriptive study, Jensen et al. highlighted how levels of mental distress were higher in patients with PFPS [26]. So far, very few studies have commented on the impact of the lockdown on knee pathologies. The study by Endstrasser et al. highlighted that the lockdown had significant consequences on pain, joint function, physical function, and physical activity in patients with overt osteoarthritis of the knee and hip [12]. Another analysis reports a high incidence of anxiety and depression among patients with PFPS [27], aspects described as among the most frequent all over the world during the lockdown [28,29,30].

Another result that emerged from our study was the increase in the indication for physiotherapy. This data is easily explained by the alleged reduction in muscle tone resulting from the sedentary lifestyle during the lockdown [16] and it is sustained by the association of other knee pathologies concurring with PFPS in our patients. Furthermore, physiotherapy was proven to be one of the most effective treatment in PFPS [31].

This study presents several limitations. First, it is a case series. Second, the medical examinations were conducted by two different orthopedic surgeons, therefore, the diagnoses and choice of treatment may have been affected by a certain degree of subjective interpretation. Third, there is still little literature about the impact of SARS-CoV-2 pandemic on knee pathologies and numerous future studies should investigate pathology changes, possibly further increasing the sample size. An interesting aspect we believe that should be explored is the study of possible changes in treatment therapies, both surgical and conservative. Another important limitation of this study is the absence of a radiological examination before and after the lockdown for all patients, which would have added an objective basis for comparison of the eventual worsening of the disease.

## 5. Conclusions

The present study suggests that the SARS-CoV-2 pandemic lockdown did not drastically change knee pathology, but it may have had an impact on it, highlighting a summary worsening of patellofemoral disorders in patients with associated knee diagnoses. Further studies are required to validate this result with more in-depth clinical and radiological elements and to evaluate any differences in surgical treatment.

## Figures and Tables

**Table 1:** Knee diagnoses groups. Groups of diagnosis related to the knee according to the etiopathogenesis and the included knee pathologies of each group.

Groups	Pathologies
1. Post-surgery follow-up	Follow-up of the patients who underwent surgery (i.e., arthroscopies, prosthetic surgery)
2. Degenerative disease	Osteoarthritis
3. Meniscus pathology	Traumatic and degenerative meniscal tears
4. Ligamentous pathology	ACL tears, PCL tears, ACL re-rupture
5. Patellofemoral disorders	Patellofemoral pain syndrome, Patellar lateralization
6. Osteocartilage pathology	Bone edema, Osteonecrosis, Osteochondritis dissecans
7. Growth pathology	Osgood-Schlatter Disease, Siding-Larsen-Johanson Syndrome
8. Traumatic injuries	Follow-up of fractures, sprains, contusions, patellar instability
9. Other causes of knee pain	Bursitis, synovitis, neoplasm, Quadriceps tendon hematoma, Intraarticular cystis, Septic arthritis, Varus/Valgus knee.
10. Unknown cause of knee pain	

**Table 2:** Characteristics of patients who underwent knee medical examination (first visits), who were diagnosed with patellofemoral disorders (group 5). Comparison of patients who were diagnosed with patellofemoral disorders in 2019 and 2020. Physical therapy as follow: (1) magneto therapy, (2) tecar therapy, (3) magneto therapy + tecar therapy. Fisher’s exact test was used to assess the differences between groups in terms of categorical variables, unpaired t tests were applied for numerical ones. The table shows the *p* value of the test. * *p* < 0.05 for the 2019 vs. 2020 group.

	2019	2020	*p* Value
**Medical records**	46	29	
**Sex** M/F	14/32	14/15	0.145
**Age** (mean ± SD)	44 ± 17.02	48 ± 17.36	0.303
**Associated diagnosis**	5	12	0.004 *
Degenerative disease	1	4	
Meniscus pathology	4	7	
Osteocartilage pathology		1	
**Additional imaging**	18	8	0.332
**Weight loss**	3	1	0.999
**Home exercises**	40	24	0.740
**Drug therapy**	3	4	0.419
**Physical therapy (1/2/3)**	9/3/5	1/4/2	0.313
**Physiotherapy**	1	6	0.012 *
**Surgery**	1	0	0.999

## Data Availability

Data supporting reported results can be found in database generated during the study.

## References

[1] Luceri F., Morelli I., Accetta R., Mangiavini L., Maffulli N., Peretti G.M. (2020). Italy and COVID-19: The Changing Patient Flow in an Orthopedic Trauma Center Emergency Department. J. Orthop. Surg..

[2] Zagra L., Faraldi M., Pregliasco F., Vinci A., Lombardi G., Ottaiano I., Accetta R., Perazzo P., D’Apolito R. (2020). Changes of Clinical Activities in an Orthopaedic Institute in North Italy during the Spread of COVID-19 Pandemic: A Seven-Week Observational Analysis. Int. Orthop..

[3] Briguglio M., Giorgino R., Dell’Osso B., Cesari M., Porta M., Lattanzio F., Banfi G., Peretti G.M. (2020). Consequences for the Elderly After COVID-19 Isolation: FEaR (Frail Elderly amid Restrictions). Front. Psychol..

[4] Perazzo P., Giorgino R., Briguglio M., Zuffada M., Accetta R., Mangiavini L., Peretti G.M. (2020). From Standard to Escalated Anticoagulant Prophylaxis in Fractured Older Adults With SARS-CoV-2 Undergoing Accelerated Orthopedic Surgery. Front. Med..

[5] Brayda-Bruno M., Giorgino R., Gallazzi E., Morelli I., Manfroni F., Briguglio M., Accetta R., Mangiavini L., Peretti G.M. (2021). How SARS-CoV-2 Pandemic Changed Traumatology and Hospital Setting: An Analysis of 498 Fractured Patients. J. Clin. Med..

[6] Borghesi M., Zilio F., Braito G., Dallago M., Muraglia S., Todaro D., Bonmassari R. (2021). How to Keep the Cath-Lab of a Hub Center “Covid Free” during the Pandemic in a Hub & Spoke Cardiology Network: A Single Center Experience and Literature Review. Minerva Cardiol. Angiol..

[7] Morelli I., Luceri F., Giorgino R., Accetta R., Perazzo P., Mangiavini L., Maffulli N., Peretti G.M. (2020). COVID-19: Not a Contraindication for Surgery in Patients with Proximal Femur Fragility Fractures. J. Orthop. Surg..

[8] Di Renzo L., Gualtieri P., Pivari F., Soldati L., Attinà A., Cinelli G., Cinelli G., Leggeri C., Caparello G., Barrea L. (2020). Eating Habits and Lifestyle Changes during COVID-19 Lockdown: An Italian Survey. J. Transl. Med..

[9] Palazzo C., Nguyen C., Lefevre-Colau M.M., Rannou F., Poiraudeau S. (2016). Risk Factors and Burden of Osteoarthritis. Ann. Phys. Rehabil. Med..

[10] Abbasi J. (2017). Can Exercise Prevent Knee Osteoarthritis?. JAMA.

[11] Zhaoyang R., Martire L.M. (2019). Daily Sedentary Behavior Predicts Pain and Affect in Knee Arthritis. Ann. Behav. Med..

[12] Endstrasser F., Braito M., Linser M., Spicher A., Wagner M., Brunner A. (2020). The Negative Impact of the COVID-19 Lockdown on Pain and Physical Function in Patients with End-Stage Hip or Knee Osteoarthritis. Knee Surg. Sports Traumatol. Arthrosc..

[13] Petersen W., Ellermann A., Gösele-Koppenburg A., Best R., Rembitzki I.V., Brüggemann G.P., Liebau C. (2014). Patellofemoral Pain Syndrome. Knee Surg. Sports Traumatol. Arthrosc..

[14] Boling M., Padua D., Marshall S., Guskiewicz K., Pyne S., Beutler A. (2010). Gender Differences in the Incidence and Prevalence of Patellofemoral Pain Syndrome. Scand. J. Med. Sci. Sports.

[15] Fulkerson J.P., Arendt E.A. (2000). Anterior Knee Pain in Females. Clin. Orthop. Relat. Res..

[16] Bowden Davies K.A., Pickles S., Sprung V.S., Kemp G.J., Alam U., Moore D.R., Tahrani A.A., Cuthbertson D.J. (2019). Reduced Physical Activity in Young and Older Adults: Metabolic and Musculoskeletal Implications. Ther. Adv. Endocrinol. Metab..

[17] Füzéki E., Groneberg D.A., Banzer W. (2020). Physical Activity during COVID-19 Induced Lockdown: Recommendations. J. Occup. Med. Toxicol..

[18] Pattyn E., Verdonk P., Steyaert A., Vanden Bossche L., Van Den Broecke W., Thijs Y., Witvrouw E. (2011). Vastus Medialis Obliquus Atrophy: Does It Exist in Patellofemoral Pain Syndrome?. Am. J. Sports Med..

[19] Cowan S.M., Bennell K.L., Hodges P.W., Crossley K.M., McConnell J. (2001). Delayed Onset of Electromyographic Activity of Vastus Medialis Obliquus Relative to Vastus Lateralis in Subjects with Patellofemoral Pain Syndrome. Arch. Phys. Med. Rehabil..

[20] Prins M.R., van der Wurff P. (2009). Females with Patellofemoral Pain Syndrome Have Weak Hip Muscles: A Systematic Review. Aust. J. Physiother..

[21] Bolgla L.A., Malone T.R., Umberger B.R., Uhl T.L. (2008). Hip Strength and Hip and Knee Kinematics during Stair Descent in Females with and without Patellofemoral Pain Syndrome. J. Orthop. Sports Phys. Ther..

[22] Baldon R.D.M., Nakagawa T.H., Muniz T.B., Amorim C.F., Maciel C.D., Serrão F.V. (2009). Eccentric Hip Muscle Function in Females with and without Patellofemoral Pain Syndrome. J. Athl. Train..

[23] Mukhtar S. (2020). Psychological Health during the Coronavirus Disease 2019 Pandemic Outbreak. Int. J. Soc. Psychiatry.

[24] Singh S., Roy D., Sinha K., Parveen S., Sharma G., Joshi G. (2020). Impact of COVID-19 and Lockdown on Mental Health of Children and Adolescents: A Narrative Review with Recommendations. Psychiatry Res..

[25] Glowacz F., Schmits E. (2020). Psychological Distress during the COVID-19 Lockdown: The Young Adults Most at Risk. Psychiatry Res..

[26] Jensen R., Hystad T., Baerheim A. (2005). Knee Function and Pain Related to Psychological Variables in Patients with Long-Term Patellofemoral Pain Syndrome. J. Orthop. Sports Phys. Ther..

[27] Domenech J., Sanchis-Alfonso V., López L., Espejo B. (2013). Influence of Kinesiophobia and Catastrophizing on Pain and Disability in Anterior Knee Pain Patients. Knee Surg. Sports Traumatol. Arthrosc..

[28] Rehman U., Shahnawaz M.G., Khan N.H., Kharshiing K.D., Khursheed M., Gupta K., Kashyap D., Uniyal R. (2021). Depression, Anxiety and Stress Among Indians in Times of Covid-19 Lockdown. Community Ment. Health J..

[29] Benke C., Autenrieth L.K., Asselmann E., Pané-Farré C.A. (2020). Lockdown, Quarantine Measures, and Social Distancing: Associations with Depression, Anxiety and Distress at the Beginning of the COVID-19 Pandemic among Adults from Germany. Psychiatry Res..

[30] Fountoulakis K.N., Apostolidou M.K., Atsiova M.B., Filippidou A.K., Florou A.K., Gousiou D.S., Katsara A.R., Mantzari S.N., Padouva-Markoulaki M., Papatriantafyllou E.I. (2021). Self-Reported Changes in Anxiety, Depression and Suicidality during the COVID-19 Lockdown in Greece. J. Affect. Disord..

[31] Heintjes E.M., Berger M., Bierma-Zeinstra S.M., Bernsen R.M., Verhaar J.A., Koes B.W. (2003). Exercise therapy for patellofemoral pain syndrome. Cochrane Database of Systematic Reviews.

